# Subcellular location and photodynamic therapeutic effect of chlorin e6 in the human tongue squamous cell cancer Tca8113 cell line

**DOI:** 10.3892/ol.2014.2720

**Published:** 2014-11-20

**Authors:** WEI LUO, RONG-SEN LIU, JIAN-GUO ZHU, YING-CHAO LI, HONG-CHEN LIU

**Affiliations:** 1 Institute and Department of Stomatology, Chinese People’s Liberation Army General Hospital, Beijing 100853, P.R. China; 2 Department of Laser Medicine, Chinese People’s Liberation Army General Hospital, Beijing 100853, P.R. China

**Keywords:** photodynamic therapy, chlorin, mitochondria, human tongue squamous cell carcinoma, cell death

## Abstract

The present study aimed to investigate the distribution and photodynamic therapeutic effect of chlorin e6 (Ce6) in the human tongue squamous cell carcinoma Tca8113 cell line *in vitro*. The distribution of Ce6 in the Tca8113 cells was observed *in situ* combined with mitochondrial and lysosomal fluorescent probes. Next, 630-nm semiconductor laser irradiation was performed. The MTS colorimetric method was used to determine cell survival. Annexin V fluorescein isothiocyanate/propidium iodide (PI) double staining was used to detect early apoptosis following photodynamic therapy (PDT). The flow cytometer was used to analyze the DNA content subsequent to PI-staining. It was observed that Ce6 could combine with the cellular membrane following 30 min of incubation with the Tca8113 cells. As the length of incubation increased, Ce6 gradually entered the cells in a particular distribution and reached saturation by 3 h. Co-localization analysis demonstrated that Ce6 was more likely to be present in the mitochondria than in the lysosomes. The cells incubated with 5 μg/ml Ce6 for 24 h exhibited a low toxicity of 5%, however, following light irradiation, Ce6-PDT was able to kill the Tca8113 cells *in vitro*. The cell toxicity was positively correlated with Ce6 concentration and light dose, therefore, the effect of Ce6 was concentration/dose-dependent (P<0.01). The lower Ce6 concentrations and light doses could significantly induce apoptosis in the Tca8113 cells, while higher doses increased necrosis/percentage of dead cells. In summary, Ce6 saturated the Tca8113 cells following 3 h of incubation. Furthermore, Ce6-PDT effectively killed the cultured Tca8113 cells *in vitro* at a safe concentration. At a low concentration and light dose, Ce6 is more likely to induce cell apoptosis via the mitochondria than the lysosomes.

## Introduction

Tongue squamous cell carcinoma (TSCC) is the most common form of malignant tumor of the oral cavity, and is ranked seventh most common amongst all cancers globally (1). TSCC accounts for ~3% of all malignant tumors. Although the incidence of TSCC is lower than that of other malignant tumors, its anatomical site is unique in that it affects the cheek, tongue, lips, palate, lower oral cavity, upper and lower jaw, and other organs. Therefore, the physical and mental impact on patients with TSCC must be addressed (2). Squamous carcinomas account for 90% of all oral and maxillofacial malignant tumors (3). While beneficial to the majority of patients, surgery, radiotherapy, chemotherapy and other traditional treatments inevitably have a functional impact on the face, head, neck and other organs. In addition, patients experience other post-treatment difficulties in facial reshaping, speech and ventilation, which have consequences for their quality of life. Therefore, there is an urgent clinical requirement to develop a safe and effective novel treatment that differs from the traditional methods.

Photodynamic therapy (PDT) for the treatment of tumors is a novel technique that has been developed in recent decades. Subsequent to injection into the body, photosensitizer selectively accumulates in tumor tissue. Upon excitation by an appropriate light source, a photochemical reaction occurs in the tumor tissues, producing reactive oxygen species (ROS) and other toxic substances that can kill tumor cells. A number of novel types of photosensitizer and corresponding laser systems have been researched and developed, with high safety and effectiveness, and minimal invasiveness. The use of such photosensitizers has therefore become popular in the field of research and development in recent years. At present, PDT has demonstrated a broad range of therapeutic applications, including the treatment of macular degeneration, skin diseases and cancer. In the present study, the photodynamic effect of the second-generation photosensitizer, chlorin e6 (Ce6), on the human TSCC Tca8113 cell line was investigated to provide an experimental basis for research in the body.

## Materials and methods

### Cell cultures and reagents

**Figure 1:**
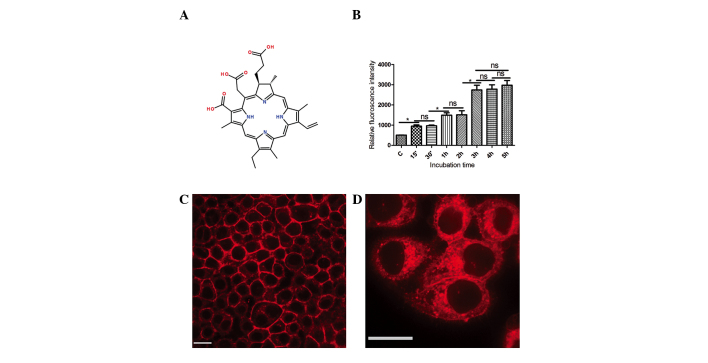
Chlorin e6 (Ce6) uptake in Tca8113 cells. (A) Molecular structure of Ce6. (B) Ce6 relative fluorescence intensity with varying incubation times. ^*^P<0.05. Fluorescence images of Ce6 distribution in Tca8113 cells following (C) 30 min and (D) 1 h of incubation (bar scale, 20 μm). Ns. no significant difference.

Ce6 was purchased from Frontier Scientific (Logan, UT, USA), and its basic molecular structure is shown in Fig. 1A. The human TSCC Tca8113 cell line was provided by the Third Military Medical University (Chongqing, China). The Tca8113 cells were cultured in Dulbecco’s modified Eagle’s medium and 10% fetal calf serum, supplemented with 100 U/ml streptomycin and 100 U/ml penicillin (all Gibco, Carlsbad, CA, USA). The trypsin and Annexin V fluorescein isothiocyanate (FITC)/propidium iodide (PI) apoptosis detection kit were purchased from Sigma (St. Louis, MO, USA). The mitochondrial (ab112143) and lysosomal (ab112137) probes were purchased from Abcam (Cambridge, UK). The MTS kit was purchased from Promega (Madison, WI, USA). The 96- and 6-well plates, 50-ml flasks and 35-mm cell culture dishes were purchased from Corning Costar (Amsterdam, the Netherlands), the 35-mm glass-bottomed culture dish was purchased from Nest (Wuxi, China) and the phosphate-buffered saline (PBS) was purchased from Serva Electrophoresis GmbH (Heidelberg, Germany).

### Method

#### Photosensitizer preparation

In sterile conditions, a 160-μg/ml stock solution was prepared by dissolving Ce6 powder in PBS at pH 7.4. This was stored at 4°C in the dark for subsequent use. For the study, the stock solution was diluted into a working solution at corresponding concentrations, as stated later.

#### Uptake of photosensitizer

In total, 10,000 Tca8113 cells were pre-treated with 40 μg/ml Ce6 for 30 min to 5 h in a glass-bottomed culture dish. The photosensitizer-containing culture medium was disposed of. Subsequent to washing twice with PBS, mitochondrial and lysosomal probes were added to the dishes. Fluorescence images were collected using a confocal laser-scanning microscope (UltraView VOX; PerkinElmer, Waltham, MA, USA). The excitation wavelengths were 405 nm (Ce6), 488 nm (mitochondrial probe) and 561 nm (lysosomal probe).

#### Laser irradiation

In total, 5,000 Tca8113 cells were seeded into the 96-well culture plates, or 6×10^5^/well in the 6-well plates/35-mm dishes. The cells were pre-treated with 0.5–20 μg/ml Ce6 for 3 h in the 37°C incubator, then washed twice with PBS and incubated for a further 30 min. The cells were then illuminated in a dark room for 5–150 sec with a LD630 semiconductor diode laser light, provided by the Laser Division of the People’s Liberation Army General Hospital (Beijing, China), to achieve total light doses of 0.5–15 J/cm^2^.

#### MTS assay

The assay was conducted according to the instructions of the MTS test kit. Subsequent to treatment with Ce6-PDT, the cells were incubated for 24 h in the 37°C incubator. In total, 20 μl MTS reagent was added to each well. After 2 h, the 490-nm absorbance values were measured using an ELX-800UV plate reader (BioTEK, Winooski, VT, USA).

#### Early apoptosis assessment by Annexin V staining

The cells subjected to PDT were detached using 2.5% trypsin (Gibco) 24 h after illumination. Early apoptosis was assayed using the Annexin V-FITC/PI apoptosis detection kit according to the manufacturer’s instructions (Sigma). A FACSCalibur flow cytometer (BD Biosciences, San Jose, CA, USA) was used for analysis of the results.

#### DNA content detection

The detection of apoptosis was based on evaluating the DNA content of the cells with the use of PI and flow cytometry. At 24h post-treatment, the cells were collected, washed twice with PBS, fixed with 70% ethanol overnight at 4°C, rinsed twice with PBS again and then stained with 25 μg/ml PI for 30 min at 4°C. The cell suspensions were analyzed with a FACSCalibur flow cytometer (BD Biosciences).

### Statistical analysis

All data are presented as the mean ± standard deviation. Statistical comparisons were performed using the Student’s t-test and one way analysis of variance using GraphPad Prism version 5.01 software (GraphPad Software, San Diego, CA, USA). P<0.05 was considered to indicate a statistically significant difference.

## Results

### Ce6 is localized in Tca8113 cells

**Figure 2:**
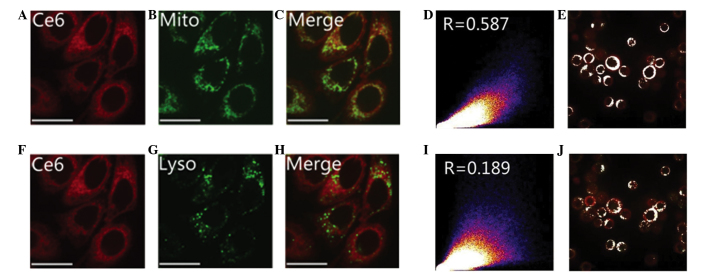
Images demonstrating Chlorin e6 (Ce6) co-localization with mitochondria and lysosomes. Following 3 h of incubation, (A and F) Ce6, (B) mitochondrial, (G) lysosomal and (C) Ce6-mitochondrial and (H) Ce6-lysosomal cellular localization was revealed via fluorescence imaging (bar scale, 20 μm). Scatter plots reveal the (D and E) Ce6-mitochondria and (I and J) Ce6- lysosome correlation coefficients. Mito, mitochondria; Lyso, lysosome.

The intensity of Ce6 fluorescence gradually increased with the extension of the co-incubation time. After 3 h, the Ce6 fluorescence intensity stabilized. No statistically significant difference was identified in the fluorescence intensity between 3, 4 and 5 h (Fig. 1B). At the beginning of photosensitizer uptake, the majority of Ce6 was located around the cell membrane, with less observed in the cytoplasm. As the incubation time increased, Ce6 was mainly distributed granularly in the cytoplasm, with little observed in the nucleus. The excitation wavelength of Ce6 was 405 nm (Fig. 1C–D). Fig. 2A–C and F–H demonstrate the cellular location of Ce6 (red), the mitochondrial and lysosomal probes (green) and the areas of co-localization (yellow) subsequent to 3 h of co-incubation. In Fig. 2C, the majority of the image appears yellow, with fewer areas of green mitochondrial fluorescence. In Fig. 2H, however, there are more areas of distinct green lysosomal fluorescence in the merged image. This demonstrates that the majority of the mitochondria, and only a small number of lysosomes, were co-localized with Ce6. The co-localization coefficient between Ce6 and the mitochondria, and Ce6 and the lysosomes was >0.5 and ~0.2, respectively. These values demonstrate that Ce6 has a higher co-localization with the mitochondria than with the lysosomes in the Tca8113 cells (Fig. 2D, E, I and J).

### Ce6 dark toxicity on human TSCC Tca8113 cells

**Figure 3:**
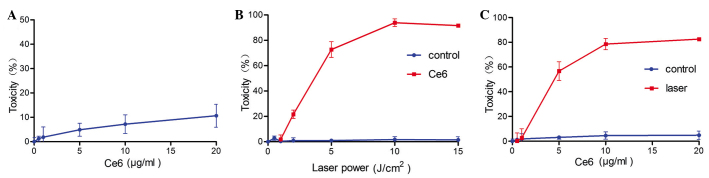
Effect of different parameters on *in vitro* Tca8113 cellular toxicity, as determined by the MTS assay. (A) Cells were cultured with varying concentrations of chlorin e6 (Ce6) for 24h in the dark, and then subjected to the MTS assay. (B) Cells in the presence (red squares) or absence (blue dots) of 2 μg/ml Ce6 were exposed to light doses of 0.5, 1, 2, 5, 10 and 15 J/cm^2^. (C) Cells were treated with 0.5–20 μg/ml chlorin e6 alone (blue dots) or in combination with a light dose of 2 J/cm^2^ (red squares). Light was applied using a 630-nm diode laser.

Using the colorimetric MTS assay, the toxicity of Ce6 on the Tca8113 cells was analyzed by measuring dehydrogenase enzyme activity. Subsequent to 24 h of incubation, 10 and 5 μg/ml chlorin-e6 induced toxicities of <10% and 5%, respectively. Considering that the Ce6 reached saturation density 3 h after incubation (Fig. 1B), the MTS data suggested that the safe drug dose for the following assays was ≤10 μg/ml (Fig. 3A), as it may otherwise lead to dark toxicity.

### Ce6 phototoxicity on Tca8113 cells

Subsequent to the MTS assay, the Tca8113 cells incubated with different concentrations of Ce6 (range, 0.5 to 20 μg/ml) and light doses (range, 0.5–15 J/cm^2^) were examined. The MTS assay demonstrated that illumination or treatment with Ce6 alone does not induce significant cellular toxicity (Fig. 3B and C). The Ce6-PDT combination treatment demonstrated almost linear correlations with light dose, Ce6 concentration and toxicity. Increasing the light dose, from 0 to 15 J/cm^2^ in the cells treated with 2 μg/ml Ce6, led to a gradual increase in cellular toxicity (P<0.05; Fig. 3B). Similarly, when the same light dose of 2 J/cm^2^ was applied, increasing the concentration of Ce6 from 5 to 20 μg/ml resulted in a gradual increase in cellular toxicity (P<0.05; Fig. 3C). A cellular toxicity of ~80% was observed following the use of 2 μg/ml Ce6 and a light dose of 5 J/cm^2^, conditions which were then applied to the subsequent experiments.

### Apoptosis detection

**Figure 4:**
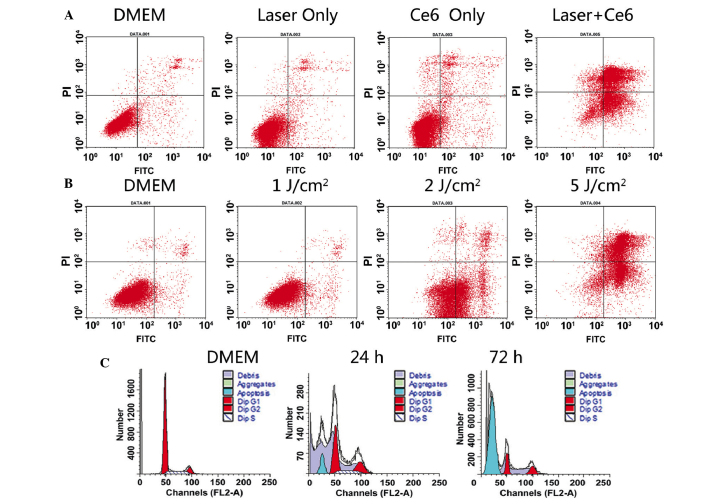
Detection of early apoptosis and DNA fragmentation. The trypsin and Annexin V fluorescein isothiocynate (FITC)/propidium iodide (PI) apoptosis detection kit was used to detect phosphatidylserine (PS) and analyze membrane integrity. (A) Chlorin e6 (Ce6) at 5 μg/ml and laser dose at 1 J/cm^2^. (B) Ce6 at 2 μg/ml and laser dose at 1, 2 and 5 J/cm^2^. As the laser dose increased, more cells underwent apoptosis and necrosis. (C) DNA fragmentation observed 24 and 72 h after PDT. For Ce6 at 5 μg/ml and a laser dose at 1 J/cm^2^, the apoptotic peaks are clearly observed. Annexin V-FITC in green fluorescence and PI in red fluorescence. DMEM, Dulbecco’s modified Eagle’s medium; FITC, fluorescein isothiocyanate.

Ce6 localized to the mitochondria and was excited by the laser to produce ROS, which triggered cell necrosis and apoptosis. It was observed that large doses of Ce6 caused necrosis, while small doses initiated apoptosis. Post-PDT cell apoptosis was detected using phosphatidylserine (PS) presentation and DNA fragmentation (Fig. 4). The apoptosis detection kit includes Annexin V-FITC and PI, and was used to detect those cells undergoing early-stage apoptosis. During the early stages of apoptosis, cells translocated to the membrane PS from the inner face of the plasma membrane to the cell surface and combined with Annexin V-FITC to produce green fluorescence, while those cells not undergoing apoptosis were not stained. PI was able to enter the dead cells and combine with the DNA to produce a red fluorescence. In the late stages of apoptosis, the integrity of the cell membrane was damaged, which enabled Annexin V and PI to enter the cells. It was revealed that illumination of Ce6-photosensitized cells led to an increase in the number of cells that expressed PS (28% in Ce6-treated cells vs. 3% in controls). Furthermore, an increase in Annexin V-PI double-positive and PI-positive cells was observed, which suggested a reduction in the integrity of the cell membrane (Fig. 4A). Similarly, increasing the light dose led to a gradual increase in Annexin V-PI double-positive and PI-positive cells, which suggested a decrease in the integrity of the cell membrane (Fig. 4B). The application of phototherapy or Ce6 alone had no observable impact on cellular apoptosis and necrosis. The presence of DNA fragmentation is a hallmark of apoptosis. In the present study, Ce6-PDT led to DNA fragmentation in ~18% and ~79% of cells, 24 and 72 h after illumination, respectively (Fig. 4C). This indicates that Ce6-PDT results in the classical apoptosis of Tca8113 cells.

## Discussion

The damage to normal tissue by PDT is mild and avoids post-operative facial destruction or functional loss, therefore, PDT has become attractive in recent years for its therapeutic potential. In 1997, the Food and Drug Administration (Silver Spring, MD, USA) listed PDT as one of the basic methods for tumor treatment, alongside surgery, radiotherapy, chemotherapy and biological immunity therapies. PDT as a treatment for cancer patients has been successful in numerous countries, including the United States, Japan, Britain, Germany, France and Canada, and has been approved by a number of countries for a variety of tumor therapies. PDT is clinically approved to treat certain types of cancers, pre-malignant conditions and macular degeneration (4–7). In addition to being effective for body surface tumors, PDT has unique advantages in treating tumors of the head and eyes, and the respiratory, gastrointestinal and urinary tracts (8–10).

The exact molecular mechanism of Ce6-based PDT is unclear. However, the production of ROS or singlet oxygen species, and the initiation of endoplasmic reticulum stress and mitochondria dysfunction, are the most common events in the process. These events are believed to lead to the induction of cell-death programs, including classical apoptosis and programmed necrosis or autophagy (11).

Photosensitizers, lasers and tissue oxygen content are three factors that enable PDT. The final effect is dependent on the photosensitizer concentration, photosensitivity, characteristic absorption spectra, extent of selective absorption of the photosensitizer in the tumor tissue, light dose and tissue oxygen supply. Ce6 is a type of chlorophyll degradation product with an absorption spectrum of 600–800 nm and a maximum absorption wavelength of 660 nm. Light-induced excitation at a long wavelength can activate the photosensitizer in deep tumor tissues to generate more efficacious treatment effects. The Ce6-PDT tissue penetration depth has been reported to reach 16.6 mm (12). In addition, Ce6 has other advantages, including a high yield of singlet oxygen, rapid clearance in the body and a short accumulation time in the skin (13).

The results of the present study suggested that Ce6 can produce a photodynamic effect on the Tca8113 cells, which increases with Ce6 concentration and light dose. The light dose and Ce6 concentration are positively correlated with PDT efficiency in cell death. Furthermore, the 24 h dark cytotoxicity of Ce6 reaches its limit at a concentration of 10 μg/ml (Fig. 3A). In addition, when Ce6 was incubated with the cells for 3 h, a period sufficient for saturation, almost no cytotoxicity was demonstrated (Fig. 3C). The experimental data revealed that a Ce6 concentration of 2 μg/ml and a light dose of 1 J/cm^2^ induced no evident Ce6-PDT-mediated effect (Fig. 3B). Similarly, a light dose of 2 J/cm^2^ and a Ce6 concentration of <1 μg/ml demonstrated almost no observable effect on cell viability. Therefore, an ideal result of cellular toxicity with 82% of cells apoptosed after 24 h can be achieved with 5 μg/ml Ce6 and a 2 J/cm^2^ laser dose, or 2 μg/ml Ce6 and a 5 J/cm^2^ laser dose. Luo and Kessell (14) demonstrated that when a certain photosensitizer was introduced to cells, the mode of cell death of either apoptosis or necrosis was functionally associated with the light dose of PDT.

Ce6-PDT has been successful in the treatment of a variety of tumors. Sheleg *et al* demonstrated that Ce6-PDT achieved ideal results in patients with metastatic malignant melanoma (15). Furthermore, it was revealed that cellular toxicity was positively correlated with increased Ce6 concentration. Low concentrations of <10 μg/ml Ce6 had no significant impact on cells, whilst at 20 μg/ml, >10% of cells were killed. Therefore, the potential systemic damage caused by Ce6 cytotoxicity should be carefully considered. Numerous studies are analyzing complex forms of Ce6 and certain carriers that increase the local drug concentration and weaken the cytotoxicity (16–18).

In the present study, a preliminary observation was made concerning the distribution of Ce6 in the Tca8113 cells. The present study revealed that Ce6 was primarily distributed in organelles in the plasma and nuclear membrane, indicating that the injury of these sub-cellular organelles is a direct cause of cellular damage. The localization of photosensitizer to the mitochondria and lysosomes is generally accepted to lead to apoptosis and necrosis, respectively. It was observed that Ce6-PDT induced rapid mitochondrial destruction (see supplementary video content online: http://www.56.com/u56/v_MTI5NDYyOTE3.html; Morphological changes of mitochondria after Ce6-PDT). The Ce6, mitochondria and lysosomes fluoresced red, green and blue, respectively. Furthermore, yellow indicated the co-localization of mitochondria and lysosomes with Ce6. The mitochondrial probe became dispersive following photo-excitation, which indicated that the mitochondria may be a sensitive target of Ce6-PDT.

In conclusion, using an *in vitro* PDT model based on human TSCCs, the present study demonstrated that the photosensitizer Ce6 may be useful in designing PDT for the treatment of TSCC. However, the pharmacokinetics and cytotoxic mechanisms of Ce6-PDT require further study in order to lay an experimental foundation for future clinical use.

## Abbreviations

Ce6: chlorin e6
PDT: photodynamic therapy
TSCC: tongue squamous cell carcinoma
PI: propidium iodide
ROS: reactive oxygen species
CLSM: confocal laser scanning microscopy
